# Vialinin A, an Edible Mushroom-Derived p-Terphenyl Antioxidant, Prevents VEGF-Induced Neovascularization In Vitro and In Vivo

**DOI:** 10.1155/2018/1052102

**Published:** 2018-02-06

**Authors:** Himangshu Sonowal, Kirtikar Shukla, Sumedha Kota, Ashish Saxena, Kota V. Ramana

**Affiliations:** Department of Biochemistry and Molecular Biology, University of Texas Medical Branch, Galveston, TX 77555, USA

## Abstract

Increased side toxicities and development of drug resistance are the major concern for the cancer chemotherapy using synthetic drugs. Therefore, identification of novel natural antioxidants with potential therapeutic efficacies is important. In the present study, we have examined how the antioxidant and anti-inflammatory activities of vialinin A, a p-terphenyl compound derived from Chinese edible mushroom *T. terrestris* and *T. vialis*, prevents human umbilical vascular endothelial cell (HUVEC) neovascularization in vitro and in vivo models. Pretreatment of HUVECs with vialinin A prevents vascular endothelial growth factor- (VEGF) induced HUVEC cell growth in a dose-dependent manner. Further, vialinin A also inhibits VEGF-induced migration as well as tube formation of HUVECs. Treatment of HUVECs prevents VEGF-induced generation of reactive oxygen species (ROS) and malondialdehyde (MDA) and also inhibits VEGF-induced NF-*κ*B nuclear translocation as well as DNA-binding activity. The VEGF-induced release of various angiogenic cytokines and chemokines in HUVECs was also significantly blunted by vialinin A. Most importantly, in a mouse model of Matrigel plug assay, vialinin A prevents the formation of new blood vessels and the expression of CD31 and vWF. Thus, our results indicate a novel role of vialinin A in the prevention of neovascularization and suggest that anticancer effects of vialinin A could be mediated through its potent antioxidant and antiangiogenic properties.

## 1. Introduction

Neovascularization is defined as the process of formation of new blood vessels from endothelial cells. Neovascularization occurs in a wide variety of physiological and pathological conditions and is essential for embryonic development, wound healing, and physiological maintenance and also for cell growth during cancer [1, 2]. Neovascularization and angiogenesis are the associated events that contribute to the cancerous development and support cancer cell growth, proliferation, and metastasis. Abnormal formation of new blood vessels supports and meets the demand of nutrient supply for the highly metabolic cancer cells and, thus, is an important pathological feature of tumorigenesis and cancer progression [3]. Thus, antiangiogenic strategies have been developed as important therapeutic approaches to prevent tumor growth and invasion to other organs [4, 5].

Several therapeutic interventions have been postulated in the recent past to target angiogenesis. Notably, inhibitors of VEGF signaling pathways have attained considerable attention in this regard, with many of these in phase III clinical trials [4, 6, 7]. Besides VEGF inhibitors, inhibitors of Notch [8, 9], PDGF/PDGFR, FGF/FGFR, Tie2/angiopoietin, HGF/MET, and RET pathways have also been identified as potential therapeutic agents to prevent angiogenesis as well as cancer growth [3, 5, 10]. Although these therapeutic strategies are found to be quite useful, many pieces of evidence suggest the development of resistance in due course of time, treatment failure in various subsets of patients, and toxic side effects on vascular cells and cardiomyocytes confine their use in the clinic. Further, the heterogeneous nature of tumor microenvironment could also contribute to the therapy failure in many cancer cases [11–15]. Therefore, it is essential to identify novel agents to control neovascularization for better therapeutic outcomes. Recently, natural plant-derived compounds and phytochemicals have attained considerable attention as alternate and effective therapies for the prevention of many disease pathologies including various forms of cancer. Some of the plant-derived antioxidants have been shown to prevent the formation of new blood vessels in vitro and in vivo studies. Further, several studies have shown that the plant-derived compounds exert synergistic effects in combination with chemotherapeutic drugs and offer better therapeutic index with minimal toxic side effects [16–18].

In this study, we have evaluated the antineovascular ability, and anti-cancer effects of a natural antioxidant vialinin A. Vialinin A is a p-terphenyl compound with antioxidant properties [19] isolated from edible Chinese mushroom *T. terrestris* [20] and *T. vialis* [19]. Although few studies report that vialinin A is a potent inhibitor of TNF-*α*, ubiquitin-specific protease (USP5), and sentrin/SUMO-specific protease 1 (SENP1) [21–26], its anticancer effects have been least investigated. Specifically, the efficacy of vialinin A in the prevention of neovascularization is not known. Here, to the best of our knowledge, for the first time, we report the effectiveness of vialinin A in the prevention of VEGF-induced neovascularization. We have shown that vialinin A prevents VEGF-induced human vascular endothelial cell (HUVEC) proliferation, migration, tube formation, and secretion of angiogenic cytokines by HUVECs by preventing the activation of NF-*κ*B. Thus, our results identify a novel therapeutic role of vialinin A in the prevention of neovascularization and suggest that this natural antioxidant could be developed as an antiangiogenic agent in cancer therapy.

## 2. Materials and Methods

### 2.1. Materials

Vialinin A (catalog number 10010519) was purchased from Cayman Chemical Company. Endothelial cell medium (ECM) (catalog number 1001) was purchased from ScienCell Research Laboratories. Phosphate-buffered saline (PBS), penicillin/streptomycin solution, and trypsin/EDTA were obtained from Invitrogen. Fetal bovine serum (FBS) was obtained from Gemini Bio-Products. 3-(4,5-Dimethylthiazol-2-yl)-2,5-diphenyltetrazolium bromide (MTT) was obtained from Sigma. Vascular endothelial growth factor (VEGF-165) and antibodies against phosphoNF-*κ*B (p65), *β*-actin, and GAPDH were obtained from Cell Signaling Technologies. Antibodies against von Willebrand factor (vWF) and CD31 were obtained from Abcam. CM-H2DCFDA was obtained from Molecular Probes, Invitrogen. A malondialdehyde (MDA) detection kit was obtained from Oxis Research. A human angiogenesis growth panel magnetic bead Milliplex kit (number HAGP1MAG-12K) and an in vitro angiogenesis assay kit (number ECM625) were obtained from Millipore. Growth factor-reduced, phenol red-free Matrigel (number 356231) was obtained from Corning. Masson's trichrome stain kit was obtained from Polysciences Inc. All other reagents and chemicals used were of analytical grade and were obtained from Sigma.

### 2.2. Cell Culture

Human umbilical vein endothelial cells (HUVECs) (catalog number 8000) were purchased from ScienCell Research Laboratories. HUVECs and bovine aortic endothelial cell lines are the most commonly used endothelial cell lines for investigations on angiogenesis. Further, unlike primary cells, HUVECs can be multiplied easily and have been well characterized by the presence of various angiogenic markers and respond well to tumor cell-released growth factors such as VEGF and FGF. Therefore, we have used HUVECs in the present study. The HUVECs were cultured in complete endothelial cell medium (ECM) (catalog number 1001) containing endothelial cell growth supplement (ECGS) and 5% FBS and 1% penicillin/streptomycin at 37°C in a humidified atmosphere of 5% CO_2_. The cells were serum starved for overnight in 0.5% serum containing ECM without ECGS followed by treatment with VEGF ± vialinin A.

### 2.3. Measurement of Cytotoxicity

HUVECs were seeded in 96-well plates at a density of 3000 cells/well and allowed to adhere overnight. The next day, the cells were serum starved overnight in 0.5% serum containing ECM without ECGS and without or with vialinin A. Subsequently, the cells were treated with VEGF (10 ng/mL) for additional 24 and 48 h, and cell viability was determined by MTT assay. 10 *μ*L of 5 mg/mL MTT solution was added to each well of a 96-well plate; after 3 h incubation at 37°C, the media was removed and the formazan crystals were dissolved in DMSO. The absorbance was recorded at 570 nm using a Synergy2 plate reader from BioTek.

### 2.4. Cell Migration Assay

HUVECs were seeded in 12-well tissue culture plates and allowed to form a confluent monolayer. The confluent monolayer of cells was then growth arrested in 0.5% FBS containing ECM. The next day, a uniform longitudinal scratch was made at the center of the monolayer with a 10 *μ*L sterile pipette tip carefully. The monolayer was washed 3x with serum-free ECM. 0.5% serum containing ECM without or with VEGF and the indicated concentration of vialinin A was then added to the wells and incubated overnight. The wells were photographed at 0 h and 18 h using an EVOS inverted microscope. Percentage migration was calculated as {(width_0 h_ − width_18 h_)/width_0 h_ × 100} and presented as a bar graph.

### 2.5. In Vitro Angiogenesis Assay

Capillary tube formation in vitro assay was performed using an angiogenesis kit from EMD Millipore following the manufacturer's instructions. Briefly, 50 *μ*L of ECM matrix solution was added to each well of a 96-well plate carefully without forming bubbles and allowed to solidify at 37°C for 1 h. Subsequently, HUVECs were seeded onto the Matrigel matrix at a density of 10^4^ cells/well in a 0.5% serum containing ECM media without or with the indicated concentrations of vialinin A and incubated for 18 h. Tube formation of the cells was photographed using an inverted microscope at 4x magnification and quantified as described earlier [27, 28].

### 2.6. Measurement of ROS and MDA Generation in HUVECs

ROS generation was measured by flow cytometry using CM-H2DCFDA dye. HUVECs were seeded in 100 mm tissue culture dishes and allowed to adhere overnight. Growth-arrested HUVECs were then treated for 18 h with VEGF (10 ng/mL) in 0.5% serum containing ECM without ECGS and without or with the indicated concentration of vialinin A. The cells were then incubated with CM-H2DCFDA for 20 min, harvested by trypsinization, and analyzed with a Flow Cytometer (BD LSRII Fortessa). Data were analyzed using Flow Jo (Treestar, Ashland, OR, USA) and represented as fold change of mean fluorescence intensity (MFI) compared to unstained control. For the analysis of MDA levels, after the indicated treatments, the cells were harvested by scrapping in ice-cold PBS containing BHT and lysed by sonication in PBS containing BHT (10 *μ*L of 0.5 M BHT/mL). Cell debris was cleared by centrifugation at 3000*g*, and the supernatant was used in the assay as directed in the protocol provided with the kit and recording absorbance at 586 nm using a spectrophotometer. Total MDA levels (*μ*M) were calculated from the standard curve and normalized to protein levels. The results are represented as fold change compared to untreated controls.

### 2.7. Analysis of Inflammatory Cytokines Secreted by HUVECs

Analysis of angiogenic cytokines secreted by HUVECs treated without or with VEGF (10 ng/mL) ± vialinin (5 *μ*M) was performed by using a human angiogenesis/growth factor magnetic bead panel kit from Millipore (number HAGP1MAG-12 K) following the manufacturer's protocol. Briefly, after 24 h of treatment of HUVECS with VEGF ± vialinin A, the media were collected by centrifugation and filtered using a 0.2 *μ*M syringe filter. The media were then frozen in −80°C and concentrated by a using a vacuum evaporator. Equal amounts of resuspended lyophilized media were then incubated with the labeled magnetic beads. After incubating overnight, the beads were counterstained with streptavidin-phycoerythrin and analyzed using a Luminex analyzer from Millipore. Data were analyzed using the *xPONENT* software, and the results are expressed as pg/mL.

### 2.8. Western Blot Analysis

HUVECs were seeded onto 100 mm tissue culture dishes at a density of 50000 cells/cm^2^ and treated as described in each experiment. The cells were then harvested by scraping, washed with ice-cold PBS, and lysed using RIPA buffer. Equal amounts of lysates were loaded and resolved on a 12% SDS gel and then transferred onto nitrocellulose membranes. 5% non-fat dried milk in TBS-T was used to block the membranes and then incubated with the specific antibodies at 4°C overnight followed by the specific secondary antibodies for 1 h at 37°C. Immunolabeling was detected using Supersignal West Pico chemiluminescent substrate from Thermo Scientific. The same membranes were reprobed by stripping with Restore Plus stripping buffer (Thermo Scientific) and developed with *β*-actin, a loading control.

### 2.9. NF-*κ*B Transcription Factor Assay

NF-*κ*B (p56) transcription factor DNA-binding assay was performed using a transcription factor assay kit from Cayman Chemicals (number 10007889) following the manufacturer's protocol. Briefly, nuclear extracts were prepared using a nuclear isolation kit, and equal amounts of nuclear extracts were loaded onto coated wells provided with the kit. The primary and secondary antibodies were added and incubated for 1 h. The developing solution was added, and the antigen-antibody complex formation was recorded at absorbance 450 nm using a Synergy2 microplate reader.

### 2.10. In Vivo Matrigel Plug Angiogenesis Assay

In vivo Matrigel plug angiogenesis assay was performed using an already established protocol [28]. 600 *μ*L growth factor-reduced Matrigel was injected into the dorsal flanks of C57BL/6J mice without or with VEGF (10 ng/mL) ± vialinin A (5 *μ*M). 10 days postinjection, the mice were sacrificed, and the Matrigel plugs were recovered. The plugs were fixed in 10% neutral-buffered formalin, and sections were cut. The sections were analyzed by staining with hematoxylin and eosin, Masson's trichrome, and antibodies for CD31 and von Willebrand factor (vWF).

### 2.11. Statistical Analysis

Data were presented as the mean ± SD (*n* = 6). Statistical analysis was carried out using a GraphPad Prism software. *p* < 0.05 is considered as statistically significant.

## 3. Results

### 3.1. Effect of Vialinin A on HUVEC Growth

**Figure 1:**
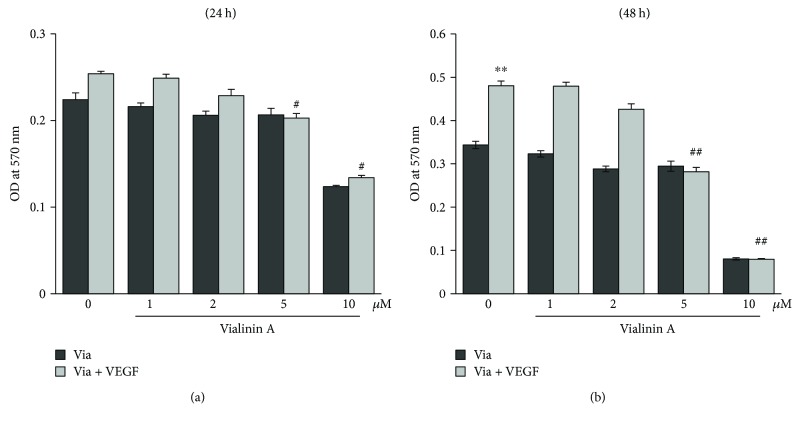
Effect of vialinin A on HUVEC proliferation. Growth-arrested HUVECs were pretreated with different concentrations of vialinin A (0 *μ*M, 2.5 *μ*M, 5 *μ*M, and 10 *μ*M) followed by treatment with VEGF (10 ng/mL) for (a) 24 h and (b) 48 h. MTT cell viability assay was performed as described in Materials and Methods. Values are the mean ± SD (*n* = 5). ^∗∗^*p* < 0.005 when compared to untreated control; ^#^*p* < 0.05 and ^#^^#^*p* < 0.005 when compared to VEGF treated.

We have first analyzed the effect of vialinin A on VEGF-induced HUVEC viability. Treatment of HUVEC with VEGF (10 ng/mL) caused a nonsignificant increase in the HUVEC cell growth after 24 h incubation, and preincubation of vialinin A prevented it. Further, at 48 h of incubation, a statistically significant (*p* < 0.001) increase in the HUVEC growth was observed in VEGF alone-treated cells (Figure 1). However, pretreatment of HUVEC with vialinin A in a concentration-dependent manner prevented the VEGF-induced HUVEC growth. Further, vialinin A alone at a concentration below 5 *μ*M did not have any effect on HUVEC cell viability at 24 h and 48 h of treatment (Figures 1(a) and 1(b)). However, at a concentration of 10 *μ*M, vialinin A alone decreased HUVEC viability. Therefore, vialinin A at a concentration of 5 *μ*M was used as an optimal level in all our studies.

### 3.2. Effect of Vialinin A on HUVEC Migration

**Figure 2:**
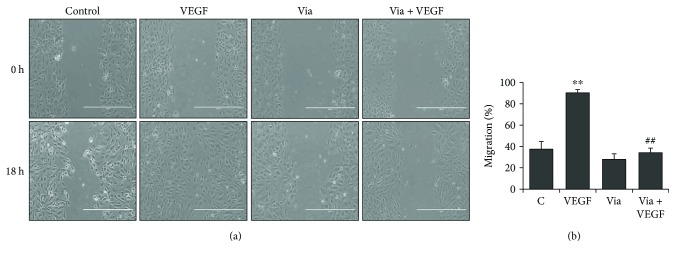
Effect of vialinin A on VEGF-induced migration in HUVEC. Growth-arrested HUVECs were pretreated with vialinin A (5 *μ*M) followed by treatment with VEGF (10 ng/mL) for 18 h. Wound scratch assay to determine cell migration was performed as described in Materials and Methods. (a) Microscopic images were taken at 0 h and 18 h showing VEGF-induced migration in HUVEC in the presence or absence of vialinin A. Scale bar = 400 *μ*m. (b) Bars showing the quantification of migration (%) as described in Materials and Methods. Representative images from three independent experiments are shown. Values are the mean ± SD (*n* = 3). ^∗∗^*p* < 0.005 when compared to untreated control; ^#^^#^*p* < 0.005 when compared to VEGF treated.

We next examined the effect of vialinin A on the VEGF-induced migration of HUVECs by a wound scratch healing assay. The data shown in Figures 2(a) and 2(b) indicate that the treatment of HUVEC with VEGF demonstrated a significant increase in the migration of HUVEC cells at the scratch sites resulting in complete closure of the wound after overnight incubation. However, treatment of HUVEC with vialinin A followed by VEGF significantly blocked the HUVEC migration. These results suggest that vialinin A prevents VEGF-induced migration of HUVECs in culture.

### 3.3. Effect of Vialinin A on HUVEC Tube Formation

**Figure 3:**
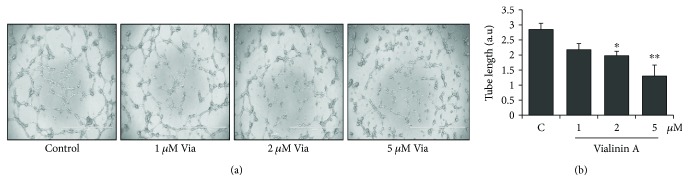
Effect of vialinin A on HUVEC tube formation in vitro. Growth-arrested HUVECs were pretreated with different concentrations of vialinin A (1 *μ*M, 2 *μ*M, and 5 *μ*M) followed by treatment with VEGF (10 ng/mL) for 24 h. In vitro Matrigel-based angiogenesis assay was performed using an angiogenesis kit from EMD Millipore following the manufacturer's instructions. (a) Images showing the effect of different concentrations of vialinin A (0 *μ*M, 1 *μ*M, 2 *μ*M, and 5 *μ*M) on growth factor-induced tube formation. Representative images from three independent experiments are shown. Magnification 4x. Scale bar = 1000 *μ*m. (b) Bars showing quantification of tube length performed by measuring individual tube length in different areas. Values are the mean ± SD. ^∗^*p* < 0.05 and ^∗∗^*p* < 0.005 when compared to untreated control.

Endothelial cell sprouting and tube formation are a significant step in the neovascularization. To examine the effects of vialinin A in the prevention of VEGF-induced neovascularization, we performed in vitro tube formation assay, a standard method to examine angiogenesis in vitro. Treatment of HUVECs with vialinin A in a dose-dependent manner prevented the HUVEC tube formation on the Matrigel matrix containing growth factors such as VEGF (Figure 3). Thus, these results indicate that vialinin A could be a potential antiangiogenic agent.

### 3.4. Effect of Vialinin A on VEGF-Induced ROS Production and Lipid Peroxidation

**Figure 4:**
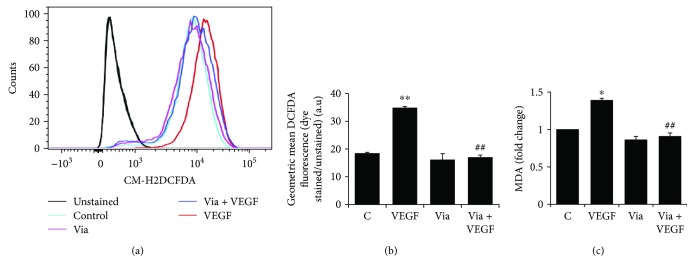
Effect of vialinin A on VEGF-induced oxidative stress in HUVEC. Growth-arrested HUVECs were pretreated with vialinin A followed by treatment without/with VEGF (10 ng/mL) overnight. The cells were stained with CM-H2DCFDA for 20 min and analyzed with a flow cytometer (BD LSRII Fortessa). (a) Histograms showing the effect of vialinin A on VEGF-induced ROS production in HUVECs (red: VEGF, blue: VEGF + vialinin A, light blue: untreated control, pink: vialinin A only, and grey solid line: unstained control). (b) Data were presented as fold change of mean fluorescence intensity (MFI) compared to unstained control analyzed using Flow Jo software. (c) MDA levels were determined with a MDA assay kit from Oxis Research. Values are the mean ± SD (*n* = 6). ^∗^*p* < 0.5 and ^∗∗^*p* < 0.005 when compared to untreated control; ^#^^#^*p* < 0.005 when compared to VEGF treated.

To examine the antioxidant efficacy of vialinin A in VEGF-induced endothelial cells, we measured VEGF-induced generation of ROS and lipid peroxidation marker malondialdehyde (MDA) in HUVECs. ROS levels were measured by staining the cells with CM-H2DCFDA followed by flow cytometry. Treatment of HUVECs with VEGF caused a significant increase in the production of ROS (Figures 4(a) and 4(b)), and preincubation of vialinin A followed by VEGF significantly prevented the formation of ROS. As compared to ROS levels in control cells, vialinin A alone treatment also reduced the formation of ROS in HUVECs. Similarly, vialinin A also prevented VEGF-induced lipid peroxidation in HUVECs. Our data shown in Figure 4(c) indicate that a significant increase in the MDA levels in the VEGF-treated HUVECs and vialinin A prevented it. These results suggest that vialinin A inhibits VEGF-induced oxidative stress in endothelial cells.

### 3.5. Vialinin A Inhibits VEGF-Induced NF-*κ*B Activation in HUVECs

**Figure 5:**
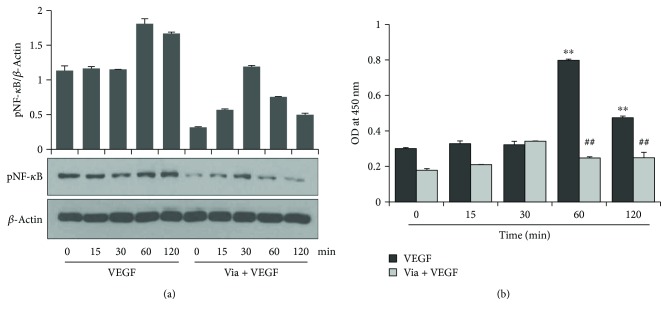
Effect of vialinin A on NF-*κ*B activation. Growth-arrested HUVECs were pretreated with vialinin A (5 *μ*M) overnight followed by treatment with VEGF for the indicated time periods. (a) Equal amounts of nuclear extracts were subjected to Western blot analysis using phospho-p65 antibodies. A representative blot is shown (*n* = 3). (b) NF-*κ*B DNA binding activity was determined by using a NF-*κ*B transcription factor assay kit from Cayman Chemicals. Data were presented as the mean ± SD (*n* = 5). ^∗∗^*p* < 0.005 when compared to untreated control; ^#^^#^*p* < 0.005 when compared to VEGF treated.

Since, NF-*κ*B is an important redox-sensitive transcription factor and regulates the transcription of inflammatory cytokines, chemokines, and growth factors; we next examined the effect of vialinin A on VEGF-induced NF-*κ*B activation in HUVECs. Our data shown in Figure 5 indicate that VEGF induced a significant increase in the phosphorylation of NF-*κ*B (p65) in a time-dependent manner in HUVEC nuclei. However, pretreatment of vialinin A followed by VEGF significantly reduced VEGF-induced phosphorylation of NF-*κ*B in the nuclear extracts of HUVECs. Similarly, as shown in Figure 5(b), NF-*κ*B transcription factor-binding assay in the nuclear lysate showed a significant increase in NF-*κ*B DNA-binding activity in VEGF-treated HUVECs and vialinin A significantly prevented it.

### 3.6. Effect of Vialinin A on VEGF-Induced Angiogenic Cytokine Production in HUVECs

We next examined the effect of vialinin A on VEGF-induced production of various inflammatory and angiogenic cytokines and chemokines in HUVECs. Treatment of HUVECs with VEGF resulted in a significant increase in the expression of angiogenic cytokines such as angiopoietin-2, follistatin, G-CSF, HB-EGF, and HGF in HUVEC cell culture media, and preincubation of vialinin A prevented VEGF-induced cytokines and chemokines (Table 1). Vialinin A alone had no significant effect on the expression of various angiogenic cytokines in HUVECs. These results suggest that vialinin A could prevent neovascularization by preventing the NF-*κ*B-mediated expression of various inflammatory and angiogenic cytokines and chemokines.

### 3.7. Effect of Vialinin A on In Vivo Angiogenesis

**Figure 6:**
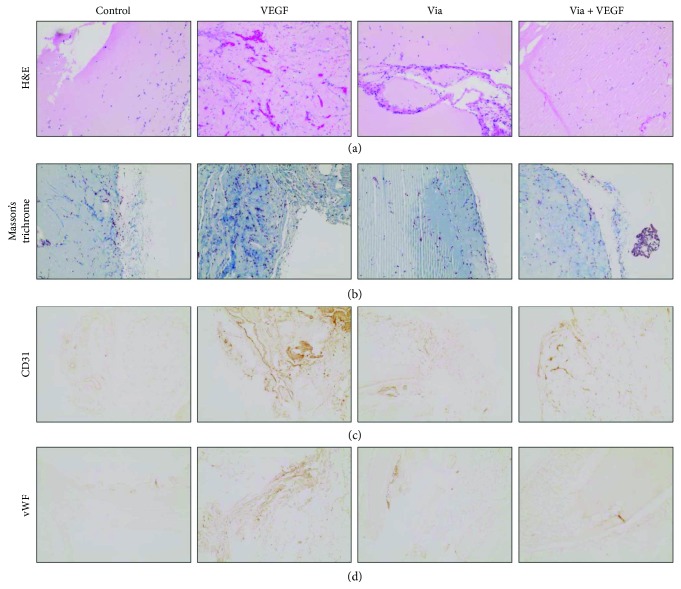
Effect of vialinin A on angiogenesis in vivo. Matrigel plugs containing vialinin A (5 *μ*M) ± VEGF (10 ng/mL) were inserted in dorsal flanks of C57BL/6J mouse. After 10 days, Matrigel plugs were dissected out, sectioned, and stained with various endothelial markers. Matrigel plug sections were stained with (a) hematoxylin and eosin, (b) Masson's trichrome, (c) CD31, and (d) von Willebrand factor (vWF). Representative images are shown (*n* = 3).

To confirm our in vitro studies, we next examined the in vivo effect of vialinin A on angiogenesis using a mouse model of the Matrigel plug method. The mice were injected with Matrigel containing VEGF (10 ng/mL) ± vialinin A (5 *μ*M). The sections of Matrigel plugs were stained for various angiogenic markers. H&E staining data shown in Figure 6(a) indicate a significant increase in the capillary-like structures in the VEGF-treated Matrigel but not in the vialinin A alone or VEGF + vialinin A containing Matrigels. Similarly, staining of sections with Masson's trichrome (Matrigel stains blue and blood vessels/endothelial cells red) suggest a significant increase in the presence of endothelial cells in VEGF-treated Matrigels but not in the vialinin A alone or VEGF + vialinin A-treated Matrigel plugs. The in vivo effect of vialinin A was further confirmed by immunocytochemical staining of Matrigel plug sections with antibodies against CD31 and vWF. The data shown in Figures 6(c) and 6(d) indicate an increase in the staining of CD31 and vWF in VEGF containing plugs, which was significantly reduced in the Matrigel plugs treated with VEGF + vialinin A. Thus, these results suggest that vialinin A prevents the formation of new blood vessels in vivo.

## 4. Discussion

Angiogenesis and neovascularization play a significant role in cancer growth and metastatic spread [29]. Signals from the tumor cells including various secreted growth factors like VEGF and FGF play a major role in triggering endothelial cells to initiate neovascularization. Thus, understanding the molecular pathways central to endothelial cell remodeling/functions will help us in designing effective therapeutic strategies for antiangiogenic therapies. VEGF has been identified as a key inducer of angiogenesis, and thereby various targeted and nontargeted therapies to disrupt VEGF-mediated tumor angiogenesis have gained attention in the recent past [5]. Several anti-VEGF treatments such as neutralizing antibodies to VEGF and VEGFRs and soluble VEGFRs have been tested in preclinical and clinical studies. Bevacizumab (anti-VEGF monoclonal antibodies) and sorafenib and sunitinib (selective VEGFRs tyrosine kinase inhibitors) have been approved by the FDA for clinical use in breast cancer, lung cancer, colorectal cancer, metastasis, and hepatocellular carcinoma. Although these drugs are given alone or in combination with other chemotherapeutic drugs, they have attained limited success owing to their unwanted side effects and off-target effects [15, 29]. To overcome these difficulties, various plant-derived and plant polyphenol-based antiangiogenic agents have gained attention in the recent past [30]. The advantages of using these plant-derived compounds and phytochemicals are their low toxic side effects and a broad spectrum of action targeting multiple signaling pathways, a multitude of which is skewed during tumor formation, angiogenesis, and metastasis [3, 31].

Vialinin A, commonly found in many edible mushroom species, through its antioxidant potential exerts anticarcinogenic effects [32]. In this study, our results demonstrate a novel antiangiogenic activity of vialinin A in vitro and in vivo. We have demonstrated that vialinin A prevents VEGF-induced proliferation as well as the migration of HUVECs. Migration and proliferation of endothelial cells are important events that initiate the process of angiogenesis. [33]. During the process of angiogenesis, endothelial cells change their morphology and sprouting of new blood vessels occurs from the existing vasculature. In response to growth factor stimuli, endothelial cells secrete components that degrade the extracellular matrix, including alterations in tight junctions, adherens junctions, and gap junctions which release the endothelial cells from the capillary intima to extravascular space and migrate to new sites and create new blood vessels [33]. Our results also demonstrate that vialinin A inhibits VEGF-induced tube formation in vitro. A significant reduction in vessel sprouting and tube formation was observed in HUVECs in vitro, which also indicates the antiangiogenic properties of vialinin A.

In addition, vialinin A also significantly reduced the endothelial cell invasion in a Matrigel plug model of angiogenesis in vivo. Hematoxylin and eosin staining of Matrigel plug sections showed a significant reduction in the number of invaded endothelial cells into the Matrigel plugs. Masson's trichrome staining also showed reduced endothelial cell invasion into the Matrigel plugs containing vialinin A. Similarly, vialinin A also prevents the expression of endothelial cell markers, CD31 and vWF, in VEGF-treated Matrigel plug sections. Thus, our results demonstrate that vialinin inhibits angiogenesis in vivo. In agreement with our results, several other antioxidants isolated from natural plants such as green tea polyphenols [34], curcumin [35–38], and resveratrol [39, 40] have been shown to be potent antiangiogenic agents with chemopreventive effects.

ROS play a significant role in maintaining the homeostasis of endothelial cell function. Further, ROS have been reported to induce proliferation of cancer cells and angiogenesis in tumors. ROS-induced activation of transcription factors has been reported to activate signaling pathways central to angiogenesis [41]. Thus, the inhibition of intracellular ROS levels is necessary to control angiogenesis as well as cancer growth and spread [42]. Several reports show that natural and synthetic compounds prevent VEGF-induced ROS and angiogenesis [17, 43, 44]. Consistent with these studies, our results also clearly demonstrate that vialinin A inhibits VEGF-induced ROS production in HUVECs. Apart from inhibition of ROS production, vialinin A also inhibits VEGF-induced generation of lipid peroxidation-derived MDA, a marker of oxidative stress. Lipid peroxidation-derived aldehydes such as HNE and MDA have been reported to induce transcription factors like NF-*κ*B and AP1 and play a significant role in regulating various inflammatory and carcinogenic signals leading to angiogenesis [28, 45]. Further, NF-*κ*B and its transcribed inflammatory cytokines and angiogenic growth factors and adhesion molecules have been shown to be involved in various forms of cancer growth and metastasis. Various natural plant-derived flavonoids and other phytochemicals have been shown to prevent angiogenesis as well as cancer growth by preventing the activation of NF-*κ*B [46, 47]. Our results also clearly indicate that vialinin A prevents nuclear translocation as well as activation of NF-*κ*B in HUVECs treated with VEGF. Thus, by inhibiting NF-*κ*B-mediated signals, vialinin A may exert its antiangiogenic functions.

Cytokines, chemokines, and growth factors such as IL-6, IL-8, IL-1*β*, TNF-*α*, TGF-*β*, GM-CSF, VEGF, and angiopoietins have been shown to promote angiogenesis and tumor metastasis [48–50]. Tumors have been reported to release a variety of inflammatory cytokines which elicit an angiogenic response in endothelial cells [51]. Growth factors trigger endothelial cell functions by producing various cytokines and chemokines which by an autocrine and paracrine manner increase the disease pathology. The agents that prevent the release of angiogenic cytokines by tumor cells and endothelial cells have been shown to be potential therapeutic agents to prevent angiogenesis as well as cancer. Our current results also demonstrate that vialinin A inhibits VEGF-induced release of various angiogenic cytokines such as angiopoietin-2, follistatin, G-CSF, HB-EGF, and HGF. Interestingly, vialinin A treatment alone also prevented the expression of some cytokines when compared to untreated control cells. The decrease in these cytokines by vialinin A alone could be due to the prevention of stress signals induced by overnight incubation of HUVECs with serum-free media. Thus, by preventing the release of various angiogenic cytokines, vialinin A could prevent VEGF-induced angiogenesis. Further studies are required to examine how inhibition of neovascularization by vialinin A affects the tumor growth and metastasis using established animal models. Completion of such studies will provide evidence that this antioxidant could be used as a cancer chemopreventive agent.

In conclusion, our results demonstrate that vialinin A prevents VEGF-induced endothelial cell proliferation, migration, and tube formation in vitro. Vialinin A also prevents VEGF-induced new blood vessel formation in a Matrigel plug model of angiogenesis in mice. Further, vialinin A inhibits VEGF-induced ROS, MDA, activation of NF-*κ*B, and production of various inflammatory and angiogenic cytokines and chemokines in HUVECs. Thus, our results suggest that vialinin A through its antioxidant and anti-inflammatory activities prevents VEGF-induced neovascularization which could be responsible for its anticancer effects.

## Figures and Tables

**Table 1:** Effect of vialinin A on VEGF-induced release of angiogenic cytokines in HUVECs.

	C	VEGF	VIA	VIA + VEGF
*Angiopoietin-2*	54869.6 ± 434.8	66142.3 ± 1184.9^∗^	25800.6 ± 373.6	26516.8 ± 179.03^##^
*BMP9*	2.6 ± 0.1	2.5 ± 0.2	2.4 ± 0.1	2.7 ± 0.5
*EGF*	306.9 ± 10.7	315.9 ± 4.3	297.2 ± 7.3	341.1 ± 19.8
*Endoglin*	5496.3 ± 211.7	4560.4 ± 317.6	3600.6 ± 45.6	3970.9 ± 172.2
*Endothelin-1*	2304.1 ± 57.4	2320.2 ± 375.4	132.4 ± 7.6	107.7 ± 7.7^##^
*FGF-1*	15.005 ± 0.5	14.6 ± 0.4	14.4 ± 0.2	14.8 ± 0.5
*FGF-2*	794.9 ± 54.5	707.3 ± 41.1	659.1 ± 22.1	751.8 ± 34.9
*Follistatin*	113.1 ± 7.2	174.3 ± 9.7^∗^	123.5 ± 2.8	157.3 ± 3.8
*G-CSF*	79.2 ± 20.4	169.1 ± 63.3^∗∗^	62.9 ± 6.6	93.7 ± 45.3^##^
*HB-EGF*	522.6 ± 20.2	1437.8 ± 38.8^∗∗^	806.1 ± 25.4	1067.1 ± 40.2
*HGF*	145.6 ± 11.4	188.5 ± 24.2^∗^	80.5 ± 3.8	97.7 ± 3.9^##^
*IL-8*	2693.04 ± 198.8	3233.3 ± 41.3	3468.1 ± 14.4	3454.6 ± 24.7
*PLGF*	5344.4 ± 23.2	5105.4 ± 79.7	2909.3 ± 11.02	2655.4 ± 64.9
*VEGF-C*	1014.4 ± 43.5	1437.1 ± 116.4^∗^	231.6 ± 12.09	262.3 ± 11.6^##^
*VEGF-D*	4.6 ± 2.7	5.4 ± 2.1	4.3 ± 1.6	5.3 ± 3.4

Growth-arrested HUVECs were treated with vialinin A (5 *μ*M) ± VEGF 10 ng/mL for 24 h. Angiogenic cytokines and chemokines were determined in the culture media using a human angiogenesis/growth factor magnetic bead panel kit from Millipore according to the manufacturer's instructions using the Milliplex system. Data shown in pg/mL analyzed by *xPONENT* software. Values are the mean ± SD (*n* = 3). ^∗^*p* < 0.5 and ^∗∗^*p* < 0.005 compared to untreated control and ^#^^#^*p* < 0.005 compared to VEGF treated.
